# The effect of age on electroencephalogram measures of anesthesia hypnosis: A comparison of BIS, Alpha Power, Lempel-Ziv complexity and permutation entropy during propofol induction

**DOI:** 10.3389/fnagi.2022.910886

**Published:** 2022-08-11

**Authors:** Daniela Biggs, Gonzalo Boncompte, Juan C. Pedemonte, Carlos Fuentes, Luis I. Cortinez

**Affiliations:** ^1^División de Anestesiología, Escuela de Medicina, Pontificia Universidad Católica de Chile, Santiago, Chile; ^2^Neurodynamics of Cognition Lab, Departamento de Psiquiatría, Escuela de Medicina, Pontificia Universidad Católica de Chile, Santiago, Chile; ^3^Programa de Farmacología y Toxicología, Facultad de Medicina, Pontificia Universidad Católica de Chile, Santiago, Chile

**Keywords:** anesthesia, age, Bispectral Index, Lempel-Ziv complexity, permutation entropy, propofol, hypnosis, EEG

## Abstract

**Background:**

Improving anesthesia administration for elderly population is of particular importance because they undergo considerably more surgical procedures and are at the most risk of suffering from anesthesia-related complications. Intraoperative brain monitors electroencephalogram (EEG) have proved useful in the general population, however, in elderly subjects this is contentious. Probably because these monitors do not account for the natural differences in EEG signals between young and older patients. In this study we attempted to systematically characterize the age-dependence of different EEG measures of anesthesia hypnosis.

**Methods:**

We recorded EEG from 30 patients with a wide age range (19–99 years old) and analyzed four different proposed indexes of depth of hypnosis before, during and after loss of behavioral response due to slow propofol infusion during anesthetic induction. We analyzed Bispectral Index (BIS), Alpha Power and two entropy-related EEG measures, Lempel-Ziv complexity (LZc), and permutation entropy (PE) using mixed-effect analysis of variances (ANOVAs). We evaluated their possible age biases and their trajectories during propofol induction.

**Results:**

All measures were dependent on anesthesia stages. BIS, LZc, and PE presented lower values at increasing anesthetic dosage. Inversely, Alpha Power increased with increasing propofol at low doses, however this relation was reversed at greater effect-site propofol concentrations. Significant group differences between elderly patients (>65 years) and young patients were observed for BIS, Alpha Power, and LZc, but not for PE.

**Conclusion:**

BIS, Alpha Power, and LZc show important age-related biases during slow propofol induction. These should be considered when interpreting and designing EEG monitors for clinical settings. Interestingly, PE did not present significant age differences, which makes it a promising candidate as an age-independent measure of hypnotic depth to be used in future monitor development.

## Introduction

The global need for surgical procedures is expected to increase in future decades, especially for older age groups (Meara et al., 2015). Elderly patients are more sensitive and more prone to postoperative adverse effects from anesthetics (Rivera and Antognini, 2009), which can have long term and life-threatening consequences (Mahanna-Gabrielli et al., 2019). Precise titration of anesthetics during surgery is key to avoid unnecessary complications arising both from excessive [e.g., longer recovery times, hypotension, and postoperative delirium (Leslie et al., 2008; Gleason et al., 2015; Goldberg et al., 2020)] and insufficient dosage [e.g., intraoperative awareness (Mashour and Avidan, 2015)]. Intraoperative use of electroencephalogram (EEG) monitors has proven to be a valuable tool in increasing the precision of anesthetic dosage (Klopman and Sebel, 2011; Punjasawadwong et al., 2014; Gao et al., 2018). However, currently commercially available monitors, like the Bispectral Index (BIS) monitor, do not consider the natural changes in a person’s EEG throughout their life (Purdon et al., 2015a), and thus may be misreading elderly patients’ depth of hypnosis. In fact, the efficacy of EEG monitors has been questioned in geriatric patients, as they have not shown advantages compared to standard treatment in some settings (Zohar et al., 2006; Yang et al., 2020). Lysakowski et al. (2009) showed that, at the time of loss of consciousness (LOC), geriatric patients presented significantly higher BIS and spectral entropy values than younger patients. Similarly, Ni et al. (2019) showed that older patients had higher BIS values despite receiving higher age-adjusted end-tidal anesthetic concentrations. A different study by Besch et al. (2011) showed that when older patients were maintained at BIS values between 40 and 60, the device’s recommended target range, they were up to 10 times more likely to be in a state of burst suppression than younger patients, suggesting overdosage. Similar systematic differences have been seen for Alpha Power (Purdon et al., 2015a), another proposed marker of states of consciousness (Purdon et al., 2013; Vijayan et al., 2013). This evidence suggests that BIS and potentially other commercially available EEG indexes based on spectral properties are affected by age (Liang et al., 2021) and could underestimate depth of anesthesia in older populations. In this context we believe it is paramount to (1) better characterize this bias to account for it in clinical settings and (2) explore the age-dependence of other candidate measures of hypnotic depth.

Recently, a family of EEG measures of hypnosis have been proposed, not based on spectral characteristics of the signal, but on information. Broadly speaking, they attempt to characterize the amount of information or entropy in the EEG signal, i.e., how chaotic vs. predictable it is in each conscious state. One of the most promising is Lempel-Ziv complexity (LZc; Lempel and Ziv, 1976) which specifically quantifies the rate at which non-redundant patterns appear in an EEG signal in time (Boncompte et al., 2021). LZc has exhibited strong dependence on different states of consciousness, showing high values during wakefulness, and low values when consciousness is lost due to a variety of anesthetics (Zhang et al., 2001), during non-REM sleep (Massimini et al., 2005) and in disorders of consciousness (Lei et al., 2022). Another information-based EEG measure that shows a strong relation with states of consciousness is permutation entropy (PE). PE is equal to the Shannon entropy of the distribution of occurrence of small patterns present in the signal (see Olofsen et al., 2008 for a detailed description). For example, if every possible pattern of activity is equally likely to occur in a signal, it will show a high permutation entropy, while if only a few patterns of activity repeatedly occur, the signal will exhibit a low PE. Similarly to LZc, PE is strongly related to subject’s states of consciousness during sleep (Hou et al., 2021), and anesthesia induced by propofol and sevoflurane (Li et al., 2008; Kreuzer et al., 2014; Liang et al., 2020). PE also shows greater values during wakefulness than after LOC. However, it remains unknown whether these measures showcase important age biases, like BIS, or if they are independent of the patient’s age.

In this study we attempted to systematically characterize the age-dependence of two spectral-based EEG markers of depth of anesthesia, BIS and Alpha Power, and to explore the possible age-dependences of two information-based EEG markers of hypnosis, LZc and PE. We did this by analyzing EEG signals from before, during and after LOC in patients of a wide age range undergoing surgical anesthesia by slow propofol infusion.

## Materials and methods

### Study design and participants

This study is reported following the STROBE (Strengthening the Reporting of Observational Studies in Epidemiology) guidelines and was registered in Clinical Trials.gov (NCT04774120). Local ethics committee approvals (Comité de Ética de Ciencias de la Salud Pontificia Universidad Católica de Chile) were obtained before data acquisition. This was a prospective cohort study conducted at the Hospital Clínico Pontificia Universidad Católica de Chile (Santiago, Chile), a tertiary medical center. Patient recruitment and informed consent were obtained during preoperative visit. Data was collected from anesthesia preoperative assessment, intraoperative electronic health records and postoperative evaluation. Patient follow-up period was up to discharge from the post-anesthesia care unit.

Our main outcome were differences in BIS, Alpha Power, LZc, and PE values (all continuous measures) between elderly (≥65 years) and young patients (18–<65 years) at LOC. Complementarily, we calculated total spectral power and spectral edge frequency during LOC (see below). LOC was defined as the time at which patients became unresponsive to verbal command, soft shaking and eyelash reflex. LOC was assessed repeatedly every 30 s until patients became unresponsive. We also calculated propofol effect-site concentration (continuous) according to Schnider’s model (Schnider et al., 1999). The start of non-zero suppression rate (yes or no) was defined as the moment when a suppression rate value [suppression rate (SR) >1%] appeared in the BIS^®^ monitor.

Eligibility criteria included patients aged >18 years, scheduled to undergo elective surgery under general anesthesia, presenting an American Society of Anesthesiologists (ASA) Physical Status I or II. Patients with preexisting neurocognitive comorbidities, use of psychoactive drugs, history of alcohol or drug abuse, body mass index greater than 35 kg m^–2^, propofol allergy, heart failure, coronary disease, or altered preoperative cognitive status (Mini-mental test <24 points) were excluded. Patients were selected from the elective surgeries schedule according to research and surgical team availability. Subsequently, they were invited to participate during preoperative evaluation. SedLine and BIS data were simultaneously obtained in the operating room.

### Anesthesia protocol

In the operating room, standard monitors (electrocardiogram, non-invasive arterial blood pressure, and pulse oximetry) were connected to the patients and a 20G peripheral venous catheter was installed for fluid and drug administration. Patients received 100% oxygen for 3 min prior to propofol infusion. Then, we administered propofol at a rate of 15 mg kg^–1^ h^–1^ in patients ≥65 years and 20–25 mg kg^–1^ h^–1^ in younger patients (<65 years) until a SR ≥1% was observed in the BIS monitor. If SR ≥ 1% was not achieved during the first 10 min after the start of propofol administration, the infusion rate was increased by 5 mg kg^–1^ h^–1^, every 5 min. Propofol infusion was stopped at SR ≥ 1% and the study protocol was considered completed at this point. Subsequently, opioids and muscle relaxants were administered, and endotracheal intubation was performed.

### Electroencephalogram-data recording and analysis

Electroencephalogram data was intraoperatively recorded using a SedLine monitor (10 μV/mm for all patients) starting minutes before propofol infusion onset until the first appearance of burst suppression as indicated by BIS monitor. Data was analyzed off-line using Python [MNE (Gramfort et al., 2013)], R and JASP (Version 0.16.3 JASP TEAM, 2022). Sampling frequency was 89 Hz for 11 out of the 12 elderly patients and 178 Hz for all other patients. We equalized the sampling frequencies of all recordings to 89 Hz to maintain consistency. Afterward, artifact-containing segments were rejected by visual inspection. Fp1 data was employed in further analyses. Excessive artifacts impeded the analysis of the baseline period. BIS values were directly obtained from the BIS monitor and averaged for each minute. To make sure the two sampling frequencies did not produce any systematic differences between age groups we did an exploratory spectral analysis at LOC of Elderly and Young patients (Supplementary Figure 1). We found that power in high frequencies (>25 Hz) was abnormally reduced in elderly patients, probably because of built-in SedLine filters. To avoid this possible confounding factor in the estimation of LZc and PE, we filtered the raw EEG signals with a butterworth lowpass filter (25 Hz, fourth order) before calculating LZc and PE. Data was segmented into 15 s segments starting at 2 min before LOC and ending at minute that showed a higher effect-site propofol concentration according to Schnider’s model (Schnider et al., 1999).

To estimate Alpha Power, we followed previously employed strategies (Şeker and Özerdem, 2016). For each 15 s segment we filtered the signal between 8 and 12 Hz (Butterworth filter, fourth order) and calculated its Hilbert transform (analytical signal). The square of the absolute value of the analytic signal yielded the spectral power within the alpha band, which we averaged across time for each 15 s segment.

Lempel-Ziv complexity was calculated using custom-made Python scripts available from previous work (Boncompte et al., 2021). Briefly, the signal from each 15 s segment was binarized across the median, assigning a 1 to all values above and a 0 to all values below the threshold, which produced the symbolic signal. Then, the LZ76 algorithm (Lempel and Ziv, 1976) was applied. This algorithm sequentially (from the beginning to the end) analyses the symbolic signal and quantifies the number of non-redundant patterns within it. Then, the number of non-redundant patterns is normalized considering the length of the signal to yield an LZc value, which ranges from 0 to 1. In general, a regular (periodic) signal showcases a low LZc (low diversity of distinct patterns) and an irregular (e.g., random) signal presents high LZc [great diversity of patterns; see Boncompte et al., 2021 for a more detailed explanation).

Permutation entropy was calculated using the “ordpy” Python library (Pessa and Ribeiro, 2021) with an embedding dimension (m) of 5 points and an embedding delay of 1. These values were chosen to follow previously published guidelines (Pessa and Ribeiro, 2021). Details of this measure can be found in previous work (Olofsen et al., 2008). Briefly, PE segments the signal into groups of m contiguous points (embedding dimension, in our case 5), and codifies the points in terms of its ordinal pattern. For example, if the 5 points sub-segment is strictly decreasing, it will be coded as {1, 2, 3, 4, 5}; if it strictly increases it will be coded as {5, 4, 3, 2, 1}; if it decreases in the first 4 points but the last one increases above all of them it will be coded as {2, 3, 4, 5, 1}. This is repeated for all sub-segments in the signal. Thus, each 15 s segment produces a high number of patterns. Permutation entropy is the Shannon entropy of this distribution of all these ordinal patterns. Shannon entropy (and PE) will be maximal for a homogeneous distribution, that is a signal in which all ordinal patterns occur with the same probability. On the other hand, Shannon entropy (PE) will be low if only a few of the possible ordinal patterns are present in the signal. Clean 15 s segments, within each minute and electrode, were averaged to obtain one value per patient per minute for each measure (PE, LZc, and Alpha Power).

Finally, to estimate Total Spectral Power and Spectral Edge Frequency 95% (SEF95), we calculated the power of each frequency component in each patient using the DFT-Welch method with hanning windows (4.5 s, 50% overlap) within the 15 s raw EEG windows. Total Alpha Power was defined as the summation of all spectral powers lower than 30 Hz. We chose this limit as our sampling frequency because it did not allow us for robust spectral estimation in higher frequencies. SEF95 was calculated from the cumulative sum of spectral powers across frequencies as the frequency value below which 95% of the spectral power was contained.

### Statistical methods

Considering a previous study reported by Lysakowski et al. (2009), a sample size of 11 subjects per group, would have 80% power to detect a difference of 15 units of BIS in the group’s averages, employing a within group standard deviations of 12 BIS units, with a significance level of 0.05 using a two-sided two-sample *t*-test.

Differences in demographic and clinical data were assessed using Independent Samples *t*-test (uncorrected, see Table 1). Normality was checked with the Shapiro–Wilk test. To analyze the effect of age on each EEG measure in different anesthetic stages, we separated our data into five 1-min segments: 2 min before LOC (LOC-2), 1 min before LOC (LOC-1), LOC, the midpoint between LOC and the maximal propofol concentration at the effect-site accordingly to Schnider’s model (LOC-CeMax) and the point of maximal effect-site concentration (CeMax). First, we conducted a *t*-test to compare each measure at LOC between groups (above or below 65 years of age). Then, for each measure (BIS, Alpha Power, LZc, and PE) we conducted a mixed-effect analysis of variance (ANOVA) with age group as the between subjects’ factor and anesthesia stage (LOC-2, LOC-1, etc.) as the within subject factor. The assumptions of sphericity of the variances were tested using Mauchly. The Huynh-Feldt correction was applied if there was violation to the sphericity assumption.

**TABLE 1:** Patients’ characteristics.

Variable	Total (*n* = 30)
Age (Mean ± SD [Range])	58.4 ± 19.1 [19–86]
Sex (%)	Male: 73,3 female: 26,7
ASA (%)	I: 23,3 II: 76,7
Charlson score (%)	0: 83,3 1: 16,7
BMI (%)	
18.5–24.9	26.7
25–29.9	53.3
30–35	20
Type of surgery (%)	
Gastrointestinal	53.3
Urological	46,7

All values are represented as mean (standard deviation) or percentage. ASA, American Society of Anesthesiologists; BMI, body mass index (kg m^–2^).

## Results

**FIGURE 1:**
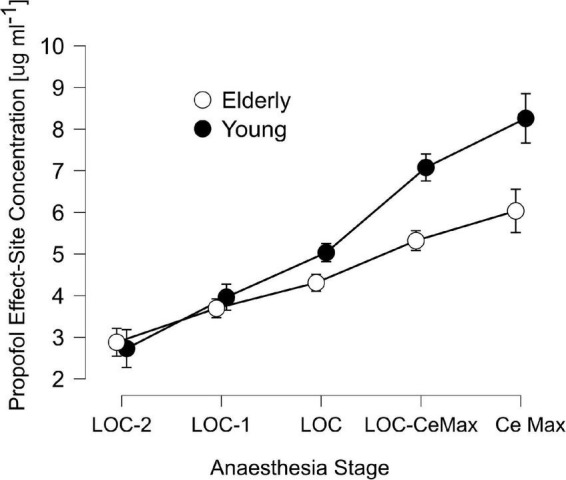
Schnider’s Effect-site propofol concentrations across anesthesia stages: 2 min before loss of consciousness (LOC) (LOC-2), 1 min before LOC (LOC-1), LOC, the midpoint between LOC and the maximal propofol concentration at the effect-site accordingly to Schnider’s model (LOC-CeMax) and the point of maximal effect-site concentration (CeMax), for elderly (unfilled circles) and young patients (filled circles). Error bars represent the 95% confidence interval.

We recruited a total of 30 patients ranging from 19 to 86 years of age that underwent urological or gastrointestinal surgery (Table 1), separated into two groups, Young (<65, median [IQR]: 44[26.5]) and Elderly patients (>65, 71.5[9.5]). Modeled effect-site propofol concentrations were similar between groups before and during LOC and deviated strongly in latter anesthetic stages (Table 2 and Figure 1).

**TABLE 2:** Induction details.

Variable	Young group (*n* = 18)	Elderly group (*n* = 12)	*p*-value
Infusion Rate [mg kg^–1^ h^–1^]	23.8 (3.1)	15.37 (0,55)	<0.001
Propofol up to LOC [mg]	128.8 (37.62)	95.64 (16,02)	0.008
Propofol Ce at LOC [μg ml^–1^]	4.82 (1.39)	4.31 (0.48)	0.237
Maximal Propofol Ce [μg ml^–1^]	6.04(1.14)	8.25(1.35)	<0.001

All values are represented as mean (standard deviation). Differences between groups were assessed with independent samples t-test.

### Age-dependence of measures of hypnotic depth

**FIGURE 2:**
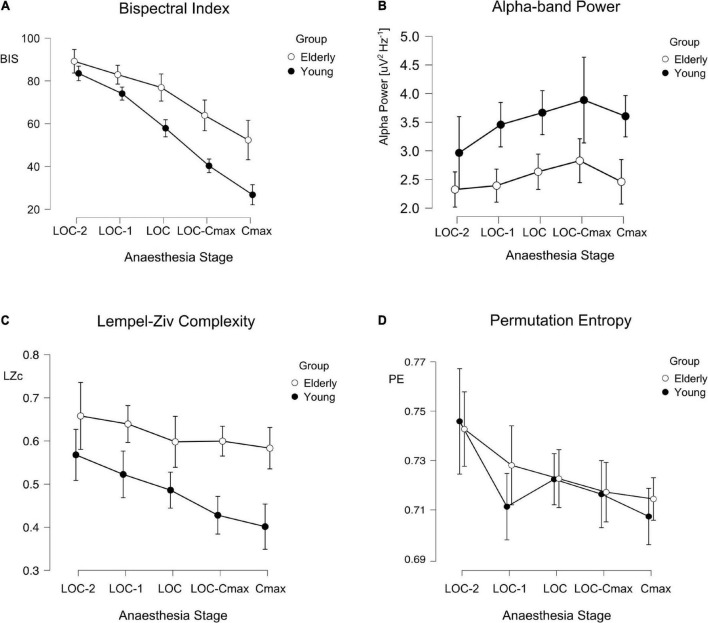
BIS, Alpha, Lempel-Ziv complexity (LZc), and permutation entropy (PE) across anesthesia stages and age groups. Line plots depicting the different values of BIS **(A)**, Alpha Power **(B)**, LZc **(C)**, and PE **(D)** across anesthesia stages for young (filled circles) and elderly (empty circles) patients: Error bars represent the 95% confidence interval.

We calculated the average value of each measure (BIS, Alpha Power, LZc, and PE) in five 1-min segments, starting 2 min before LOC and ending in the minute of maximal effect-site propofol concentration according to Schnider’s model (Schnider et al., 1999). We conducted mixed-effect ANOVAs for each hypnotic measure with age group (Young/Elderly) as the between subject factor and sedation stage as the within subject factor. These analyses showed that BIS, LZc, and PE were consistently dependent on anesthesia stage (Figures 2A,C,D; anesthesia stage effect: BIS F(2.51) = 134.0, *p* < 0.001, ω^2^ = 0.676; LZc F(3.35) = 7.54, *p* < 0.001, ω^2^ = 0.13; PE F(2.77) = 6.80, *p* < 0.001, ω^2^ = 0.098). Alpha Power did not show a significant sedation stage effect (F(2.14) = 2.35, *p* = 0.101, ω^2^ = 0.02). Alpha Power initially increased, and then decreased in latter stages of anesthesia (e.g., CeMax, see Figure 2B). BIS, Alpha Power, and LZc showed significant differences between young and elderly patients age group effects (BIS F(1) = 24.68, *p* < 0.001, 173 ω^2^ = 3.0; Alpha Power F(1) = 5.48, *p* = 0.028, ω^2^ = 0.08; LZc F(1) = 24.2, *p* < 0.001, ω^2^ = 0.317.) BIS and LZc showed greater values for elderly than for young patients while the inverse was observed for Alpha Power (Figure 2). Complementary, unpaired Student *t*-tests showed that these three measures presented significant differences at the time of LOC (Table 3, uncorrected). Interestingly PE was not significantly different between young and elderly patients [PE F(1) = 0.216, *p* = 0.646, ω^2^ < 0.001]. In addition, and as expected elderly patients showcased significantly lower total spectral power and higher SEF95 than younger populations.

**TABLE 3:** Hypnotic measures at loss of consciousness (LOC).

Measure (mean ± SD)	Young	Elderly	*p*-value
BIS	59.21 (12.58)	76.93 (15.49)	0.002
Alpha Power (μV^2^ Hz^–1^)	3.82 (1.52)	2.68 (0.70)	0.022
LZc	0.488 (0.099)	0.598 (0.119)	0.011
PE	0.721 (0.033)	0.724 (0.024)	0.816
Total Power (μV^2^)	364.5 (246.5)	59.3 (16.5)	< 0.001
SEF95 (Hz)	12.1 (5.0)	16.9 (2.3)	0.005

All values are represented as mean (standard deviation). Differences between groups were assessed with independent samples t-test.

## Discussion

In the present work we systematically analyzed the age differences of four different EEG-derived measures of hypnotic depth before, during and after loss of consciousness by continuous propofol infusion. We found that BIS, Alpha Power, and LZc displayed a significant difference between age groups throughout anesthesia stages. Elderly patients showed significantly greater BIS and LZc values and significantly lower Alpha Power. In contrast, PE did not show significant differences associated with age.

BIS is a highly employed index to quantify depth of hypnosis. Its use has been shown to reduce dosage and adverse effects of anesthesia, however, its effectiveness in older populations is contentious. We found that BIS values at LOC were on average ∼18 points higher for patients older than 65 years. Our results are consistent with previous works (Zohar et al., 2006; Lysakowski et al., 2009; Besch et al., 2011; Ni et al., 2019; Yang et al., 2020) that suggested that BIS has an age bias, specifically underestimating depth of anesthesia in patients older than 65 years of age. This population became behaviorally unconscious at higher BIS score when compared to younger patients. This is of great clinical importance, as older populations are at the greatest risk of anesthesia induced adverse effects like longer recovery times, hypotension, and postoperative delirium (Leslie et al., 2008; Gleason et al., 2015; Goldberg et al., 2020). This implies that, if BIS is employed in surgical settings on elder patients, it should not be interpreted in the same way as it is interpreted in younger populations (e.g., maintenance values between 40 and 60).

Alpha Power also showed systematic differences at LOC between age groups, with older patients presenting lower spectral power than young ones. This is consistent with previously reported decrease of spectral power within these frequencies in general in older populations (Purdon et al., 2015a; Voytek et al., 2015). Alpha Power did not show a significant main effect of anesthesia stage [F(2.14) = 2.35, *p* = 0.101, ω^2^ = 0.02], probably because of the lack of a linear dependence to anesthesia stages: Alpha Power initially increase, and then decreases in latter stages of anesthesia (e.g., CeMax, see Figure 2B) as pointed out by previous evidence (Purdon et al., 2015b). In this line, we observed a tendency toward a reduction of Alpha in the last induction stage (CeMax). This is to be expected in deeper anesthesia stages when delta waves take over alpha waves as hypnotic depth increases (Purdon et al., 2015b). Interestingly, complementary analyses of the whole frequency range (Supplementary Figure 1) showed that Alpha Power was not the only spectral band to be lower in elderly patients than in younger ones during LOC. Delta and theta bands, but not beta, were significantly lower in the Elderly group (Supplementary Figure 1). This is to be expected. It has been shown that, in general, spectral power decays with age (e.g., Purdon et al., 2015a), which can also represent changes in 1/f spectral power decay (Voytek et al., 2015).

Both entropy-related measures (LZc and PE) showed a consistent dependency on anesthesia stages, however they differed in their relation to age. LZc displayed statistically significant differences between age groups throughout induction stages (Table 3 and Figure 2) indicating an age bias: LZc underestimated the depth of hypnosis in elderly patients. Interestingly, similar age-related differences have been found during wakefulness: Mendez and cols. report greater LZc in elderly populations compared to younger ones during resting state (Mendez et al., 2012). On the other hand, PE was the same for both age groups. This contrasts with reports that Sample Entropy (a measure similar to PE) was greater in elderly patients during sleep (Bruce et al., 2009). Similarly, but in an anesthesia setting, (Kreuzer et al., 2020), reported greater PE for older patients undergoing sevoflurane surgical anesthetic administration. These differences could be explained by the parameters used in PE calculations (*m* = 5 vs. *m* = 3), sampling time points (60 vs. 10 s), sampling frequency, anesthetic timing (induction vs. maintenance), or anesthetic employed. It is important to remark that LZc and PE, although both are entropy-related measures, and are thought to be related to the same underlying factors [as 1/f slope (Medel et al., 2020)], they quantify very different aspects of a signal. PE quantifies the diversity (entropy) of very small ordinal patterns of activity; in this work those patterns lasted ∼56 ms. LZc also qualifies the diversity of patterns, however these patterns are very different. The signal is first binarized, then only patterns that are strictly non-redundant between them are selected, which can have different lengths, however, these are in general much longer than those considered in PE calculations. The differences in the age-dependency of these two measures reported here could inform further research into the specific aspects of anesthesia EEG signals that change during aging.

Limitations of this study include a relatively small sample size. Future studies with greater sample size replicating our results would be needed before practice modification. Importantly, this type of studies should attempt to record EEG using higher and homogeneous sampling frequencies, in particular when estimating PE and LZc, to avoid the need of pre-filtering the EEG signals. We based our analysis on anesthesia induction, meaning that our observations were based on non-steady state conditions. This could have affected the BIS results more noticeably, as we did not compensate for delays in calculating the index (Eleveld et al., 2018). Future studies should analyze these EEG parameters in anesthesia maintenance and during the recovery period. Also, we used clinical measures for LOC assessment trying to mimic what is usually applied in clinical practice. These clinical methods might not be as accurate as others used in more controlled settings (Purdon et al., 2013). Another consideration in the current results is that propofol infusion rates were slower in elderly patients assuming propofol requirements are lower in this group. The slower infusion rates in this group resulted in comparable times to achieve LOC in both groups and likely influenced the Ce of propofol at LOC which was similar in both groups (see Table 2). The dosing scheme used reflects clinical practice whereby elderly patients receive less propofol than younger patients at LOC. This, in combination with the fact that BIS and LZc showcased higher values in elderly patients could imply that these measures are more closely related to predicted effect-site propofol concentrations than to depth of anesthesia, however this should be tested specifically in a separate study.

In conclusion, our results showcase the presence and magnitude of the age bias of currently employed EEG derived hypnotic depth measures (BIS and Alpha band power) throughout five different induction stages. We also show that LZc also presents strong age biases during propofol induction. Importantly, these biases do not preclude the use of any of these indexes as measures of depth of hypnosis but encourage the inclusion of age as a covariable when using them to estimate depth of hypnosis in clinical settings. Importantly, because of a lack of age-dependency, PE appears as a promising candidate to be considered as an age-independent measure of hypnotic depth.

## Data Availability statement

The data is available upon reasonable request from the corresponding author.

## Ethics statement

The studies involving human participants were reviewed and approved by Comité de Ética de Ciencias de la Salud Pontificia Universidad Católica de Chile. The patients/participants provided their written informed consent to participate in this study.

## Author contributions

DB and LC: study design. DB, CF and JP: acquisition of data. DB and GB: data analysis. DB, GB, JP, and LC: interpretation of results and drafting of the manuscript. All authors contributed to the article and approved the submitted version.
