# Supplementary figures and images for: The effect of age on electroencephalogram measures of anesthesia hypnosis: A comparison of BIS, Alpha Power, Lempel-Ziv complexity and permutation entropy during propofol induction
Source: Front Aging Neurosci. 2022 Aug 11;14:910886. doi: 10.3389/fnagi.2022.910886 (PMC9404504; doi:10.3389/fnagi.2022.910886)

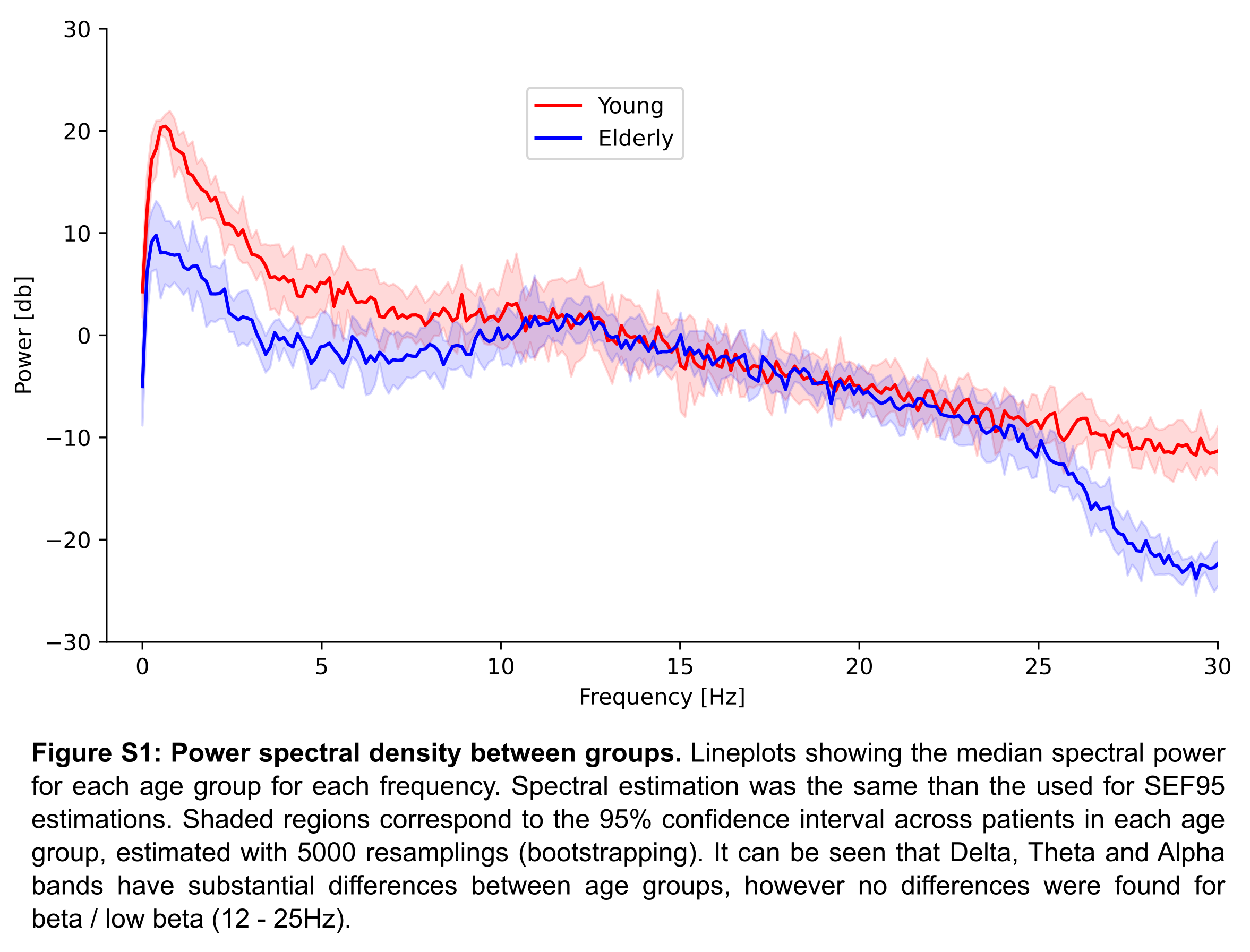

Supplement: Supplementary file 1 [file Image_1.PNG]
